# Full-length genome and molecular characterization of dengue virus serotype 2 isolated from an imported patient from Myanmar

**DOI:** 10.1186/s12985-018-1043-2

**Published:** 2018-08-20

**Authors:** Zhaoping Zeng, Jiandong Shi, Xiaofang Guo, Ling Mo, Ningzhu Hu, Jing Sun, Meini Wu, Hongning Zhou, Yunzhang Hu

**Affiliations:** 1 Institute of Medical Biology, Chinese Academy of Medical Sciences and Peking Union Medical College, Kunming, 650118 China; 2 Yunnan Key Laboratory of Vaccine Research and Development of Severe Infectious Disease, Kunming, 650118 China; 3 Yunnan Provincial Center of Arborvirus Research, Yunnan Provincial Key Laboratory of Vector-borne Diseases Control and Research, Yunnan Institute of Parasitic Diseases, Pu’er, 665000 Yunnan China

**Keywords:** Dengue virus, YNPE2 strain, Complete genome, Molecular characterization, 5′/3′ RACE

## Abstract

**Background:**

Dengue is the most common mosquito-borne infection worldwide and a serious threat to global public health. Sporadic dengue virus serotype 2 (DENV-2) imported cases from Myanmar have been documented almost every year in Yunnan Province of China since 2005. However, the complete genome sequences of DENV-2 isolates imported from Myanmar are not available.

**Methods:**

The full-length genome of the DENV-2 strain (YNPE2), isolated from an imported case from Myanmar in 2013, was identified by the next-generation sequencing. The extreme ends of the viral genome were validated by 5′/3′ RACE and Sanger sequencing. Furthermore, phylogenetic, recombination and selection pressure analyses were conducted for the molecular characterization of YNPE2 strain.

**Results:**

Whole-genome sequencing revealed that the full-length sequence of YNPE2 strain was 10,724 bases, with an open reading frame encoding for 3391 amino acids. The YNPE2 strain had 99.0% nucleotide identity and 99.8% amino acid identity with two closely related strains, ThD2_0078_01 strain (DQ181797) and DENV-2/TH/BID-V2157/200 strain (FJ639832). The phylogenetic analysis suggested that the YNPE2 strain belonged to Asian I genotype and was likely derived from Thailand strain (DQ181797). Moreover, selection pressure analysis revealed two amino acid sites of the NS4B and NS5 proteins, with important evidence of positive selection.

**Conclusion:**

This study revealed the first complete genome sequence and molecular characterization of a DENV-2 strain (YNPE2) isolated from an imported case from Myanmar, thus providing a valuable reference genome source for future surveillance, epidemiology and vaccine development of DENV-2 virus in Yunnan, China.

**Electronic supplementary material:**

The online version of this article (10.1186/s12985-018-1043-2) contains supplementary material, which is available to authorized users.

## Background

Dengue, a re-emerging mosquito-borne human viral disease, is caused by the dengue virus (DENV). The global prevalence of the disease has markedly increased in recent decades, with about half of the world′s population under threat [1]. More than 390 million people worldwide are infected by DENV every year, of which 96 million have clinical symptoms, ranging from mild dengue fever (DF) to dengue hemorrhagic fever (DHF) or Dengue shock syndrome (DSS) [2].

DENV is a single stranded and positive polarity RNA virus with a genome of about 11,000 bases in length [3], and belongs to the genus *Flavivirus*, family *Flaviviridae* [4]. The viral genome only has a long open reading frame (ORF) that encodes a polyprotein, and is flanked by 5′ untranslated region (UTR) and 3′ UTR. The polyprotein can be processed into three structural proteins (capsid, membrane and envelope) and seven non-structural (NS) proteins (NS1, NS2A, NS2B, NS3, NS4A, NS4B and NS5) [3]. The DENV is mainly transmitted by *Aedes aegypti* mosquitoes in tropical and sub-tropical countries [5]. Serologically, DENV is classified into four serotypes (DENV-1 to DENV-4), which share 65–70% homology [6]. Due to its high mutation rate, DENV has generated different genotypes and lineages within each serotype [7]. Revealing the genotype of the circulating DENV in epidemic areas is critical to disease surveillance, epidemiology and vaccine development.

With the increase in population mobility, the DENV and mosquitoes can quickly travel by modern transportation across the globe. Thus, the endemic areas of dengue have expanded from 9 countries before 1970 to over 100 countries worldwide [1]. Among them, South-East Asia, America and the Western Pacific regions are seriously affected by dengue, due to their tropical and sub-tropical climate. In South-East Asia, all countries have reported outbreaks of dengue since 1950 [8–14]. In China, the first dengue epidemic was documented in Guangdong Province in 1978 [15]. In recent decades, the number of dengue patients have gradually increased in some provinces of China, including Guangdong, Yunnan, Fujian and Zhejiang [16–19]. Generally, numerous dengue outbreaks in China are closely related to imported cases from countries in the South-East Asia. Thus, dengue remains an imported disease in China [20].

Yunnan Province is located in the Southwest border of China, and shares a 4060-km border with three dengue-endemic countries: Myanmar, Laos and Vietnam. With the construction of the China-ASEAN Free Trade Area and the transport infrastructure in the Greater Mekong Subregion, Yunnan and South Asia and South-East Asia countries are experiencing an increase in trade and tourism. Hence, Yunnan Province faces an increasing need for dengue prevention and control due to numerous imported cases from the neighboring countries. The national dengue surveillance program was implemented in Yunnan province in 2005, and an increasing number of imported cases have been reported each year [21, 22]. In 2008, an outbreak of dengue was caused by imported cases from Myanmar [21]. Moreover, a large-scale dengue outbreak was reported in 2013 in Dehong and Xishuangbanna Prefectures of Yunnan Province. The outbreak was predominantly related to imported cases from Myanmar and Laos [23]. Therefore, imported cases from Myanmar and Laos were likely the main cause of the dengue outbreak in Yunnan Province. However, majority of the previous studies on the imported DENV-2 strain were based on partial sequencing data of the envelope (E) gene. The complete genome sequence of any DENV-2 strain imported from Myanmar is not available. In this study, we reported full-length genome sequence and molecular characterization of the YNPE2 strain isolated from an imported case from Myanmar. This study will likely facilitate future surveillance, epidemiology studies and vaccine development of DENV-2 virus in Yunnan, China.

## Methods

### Case presentation

In September 2013, a 32-year-old female patient, who came from Myanmar, was clinically diagnosed with dengue fever in Ruli, Yunnan, China. She had dengue-like symptoms including fever, joint pain, myalgia and headache. The patient′s acute serum was positive for NS1 antigen by One Step Dengue nonstructural protein (NS) 1 RapiDipTM InstaTest (Cortez). The patient’s acute serum (2–5 ml) was collected with informed consent and stored at -80 °C until testing.

### Virus isolation, identification and serotyping

Acute-phase serum samples collected from the dengue patient were used for virus isolation. Briefly, the serum was diluted at a ratio of 1:10 using RPMI 1640 medium (Biological Industries, USA), followed by incubation for 1 h with *Aedes albopictus* C6/36 cells for virus adsorption in a 75 cm^2^ flask (Corning, USA). Then, RPMI 1640 medium supplemented with 2% fetal bovine serum (FBS; Gibco) was added to maintain cell survival. Cells were incubated at 28 °C in 5% CO^2^ and examined daily for cytopathic effects (CPE). When CPE was observed, the cell culture supernatant was collected, which served as virus stock. The virus stock was used for infecting C6/36 cells. Once CPE was observed, the cell culture supernatant was collected and analyzed by reverse transcription PCR (RT-PCR) to determine the serotype of the virus. The viral RNA was extracted from infected cell culture supernatant using TRIzol reagent (Tiangen, Beijing, China) according to the manufacturer′s protocol. Total RNA was reverse transcribed using GoScript™ Reverse Transcriptase (Promega, WI, USA) according to the manufacturer′s instructions. The reaction was conducted for 5 min at 25 °C, followed by 1 h at 42 °C and 15 min at 70 °C in a BioRad C1000 Cycler system. Then, PCR was performed using Taq polymerase (TaKaRa, Dalian, China) using a reference protocol with DENV universal primers (D1: 5′ -TGAATATGCTGAAACGCGCGAGAAACCG-3′ and D2: 5′ -TTGCACCAACAGTCAATGTCTTCAGGTTC-3′) and DENV-2 typing primer (D1 and TS2: 5′ -CGCCAGAAGGGCCATGAACAG-3′) [24]. Agarose gel electrophoresis was conducted to check the specificity of amplicons [24].

### Whole-genome sequencing analysis

The viral RNA was extracted from infected cell culture supernatant using the TRIzol reagent (Invitrogen, CA, USA) according to the manufacturer′s protocol. The total RNA was depleted of rRNA with an epicentre Ribo-ZeroTM kit (Illumina, CA, USA). The rRNA-depleted RNA was purified and fragmented using a RNA fragmentation kit (Ambion, USA). The sequencing library was constructed as previously described [25]. The sequencing was performed on an Illumina Hiseq 4000 platform (LC Sciences, Hangzhou, China) according to the manufacturer′s standard protocol, and 2 × 150 bp paired-end reads (PE150) were generated. The joint and low quality reads were removed from the raw sequencing reads to obtain the clean reads. The viral genome was assembled from the clean reads by the SPAdes (v3.10.1) program [26].

### 5′ / 3′ rapid amplification of cDNA ends (RACE)

The viral RNA was extracted from infected cell culture supernatant using the TRIzol reagent (Invitrogen, CA, USA). Since the total RNA was extracted from a non-eukaryotic organism and lacked a polyadenylated tail, the 5′-first-strand cDNA was synthesized with random primers and the 3′-first-strand cDNA was synthesized after adding a poly(A) tail using Poly(A) Polymerase (TaKaRa, Dalian, China) according to the instruction of SMARTer 5′/3′ RACE kit (Clontech, CA, USA). The 5′/3′ RACE PCR was performed by using SeqAmp DNA Polymerase (TaKaRa, Dalian, China) with Universal Primer Mix and 5′ Gene-Specific Primer (5′-CGCCATTATGGTGAAGCCTGGATGTCTC-3′) / 3′ Gene-Specific Primer (5′-ATCTGGGAGGCCACAAACCATGGAAGC-3′) according to the instruction of SMARTer 5′/ 3′ RACE kit. The PCR reaction involved 40 cycles of 94 °C for 30 s, 60 °C for 30 s, and 72 °C for 2 min in a BioRad C1000 Cycler system. The PCR products of 5′ RACE and 3′ RACE were diluted at a ratio of 1:10 with nuclease-free water. The second round PCRs were performed in a reaction mixture of 50 μL containing 5 μL of 10 × La Taq Buffer (Mg^2+^ Plus), 8 μL of dNTPs (2.5 mM), 5 μL of 10 × Universal Primer Mix, 1 μL of 5′/3′ Gene-Specific Primer, 2.5 μL of diluted 5′/3′ RACE PCR products and 1 unit of high fidelity La Taq DAN polymerase (TaKaRa, Dalian, China). The second round PCRs were conducted under conditions of one cycle of 94 °C for 5 min followed by 40 cycles of 94 °C for 30 s, 60 °C for 30 s, and 72 °C for 2 min. The amplified products were purified from 1.5% agarose gel using the Wizard SV Gel and PCR Clean-Up System (Promega, WI, USA). The purified products were ligated to the T4 vector (Promega, WI, USA), transformed into E. coli, and positive clones were selected for Sanger sequencing (BGI, Shenzhen).

### Accession number

The full-length genome sequence of the DENV-2 strain YNPE2 was deposited in GenBank (accession number MF459663.3).

### Genomic characterization analysis

The viral genomic characterization and amino acid sequence was analyzed using the DNASTAR Lasergene 11.1 software package (DNASTAR Inc., USA). Multiple sequence alignments were performed using the ClustalX2.1 program [27]. The Mfold software package (http://unafold.rna.albany.edu/?q=mfold) was utilized to predict the secondary structure of the UTRs of the viral genome with the default folding parameters [28].

### Phylogenetic analysis

Phylogenetic analysis based on the DENV-2 coding sequence and the envelope gene were performed using the MEGA program (version 6.06) [29, 30]. The phylogenetic trees were deduced by employing the Maximum Likelihood (ML) method based on the General Time Reversible model [31]. The bootstrap replications was set at 1000 for estimating the Branch topology of the phylogenetic tree.

### Recombination analysis

Recombination detection of the DENV-2 was conducted by seven methods, including RDP [32], Chimaera [33], BootScan [34], 3Seq [35], GENCONV [36], MaxChi [37] and SiScan [38] available in the Recombination Detection Program (RDP version 4.95), with *p* < 0.01. The recombinant event was identified if a sequence was detected by at least three methods under the multiple comparison correction setting option.

### Selection pressure analysis

The selection pressure analysis for codons of the open reading frame (ORF) of the viruses (excluding potential recombinants) was performed by single-likelihood ancestor counting (SLAC) [39], fixed effects likelihood (FEL) [40], internal fixed effect likelihoods (IFEL) [41] and mixed effects model of evolution (MEME) [42] available in HyPhy (Hypothesis testing using phylogenies) open-source software package (http://www.hyphy.org/). The ratio of non-synonymous to synonymous substitutions (dN/dS ratio, ω) were calculated in four methods. The sites were considered significant if they were detected by three or more methods.

## Results

### Identification and serotyping of virus

**Fig. 1:**
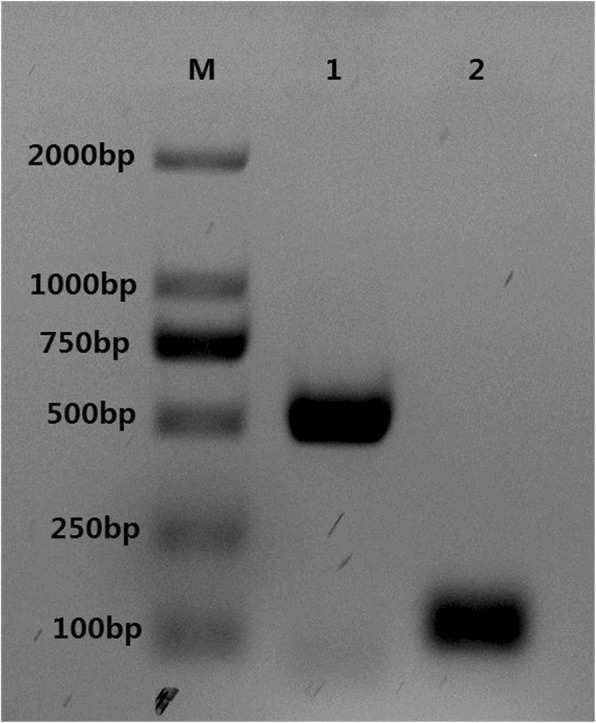
The new DENV-2 isolate identified by RT-PCR. **M:** DNA marker; **1:** a 511-bp nucleotide fragment was obtained after amplification with DENV universal primers; **2:** a 119-bp nucleotide fragment was obtained after amplification with DENV-2 type-specific primers

To observe the proliferation properties of the virus, C6/36 cells were repeatedly infected with virus stock. At 8 days post-infection, typical CPE was observed in the C6/36 cells. Furthermore, the RT-PCR analysis showed two specific bands, 511 bp and 119 bp in length, which were specifically amplified by DENV universal primers and DENV-2 typing primers (Fig. 1), indicating that the virus was dengue virus serotype 2. The virus was named as YNPE2 strain.

### Full-length sequence and genomic characterization of YNPE2

**Fig. 2:**
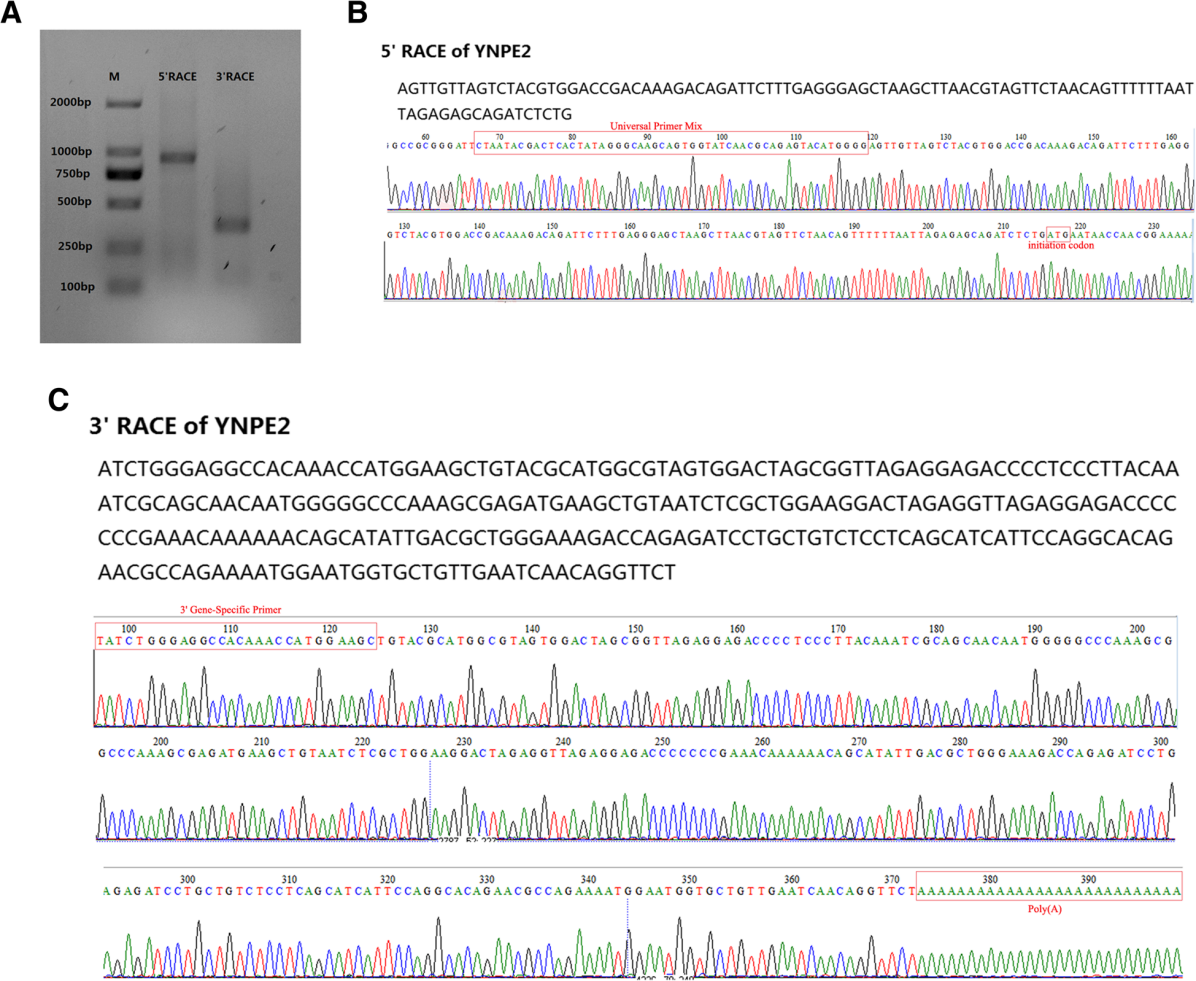
The 5′/3′ RACE PCR and Sanger sequencing results of YNPE2. **a** The gel shows the 5′ RACE and 3′ RACE amplifications of YNPE2. **M:** DNA marker; **5′ RACE:** a 849-bp nucleotide fragment was obtained after amplification with Universal Primer Mix and 5′ Gene-Specific Primer; **3′ RACE:** a 275-bp nucleotide fragment was obtained after amplification with Universal Primer Mix and 3′ Gene-Specific Primer. **b** the Sanger sequencing result of 5′ RACE, only part of the sequencing result is displayed; **c** the Sanger sequencing result of 3′ RACE

**Fig. 3:**
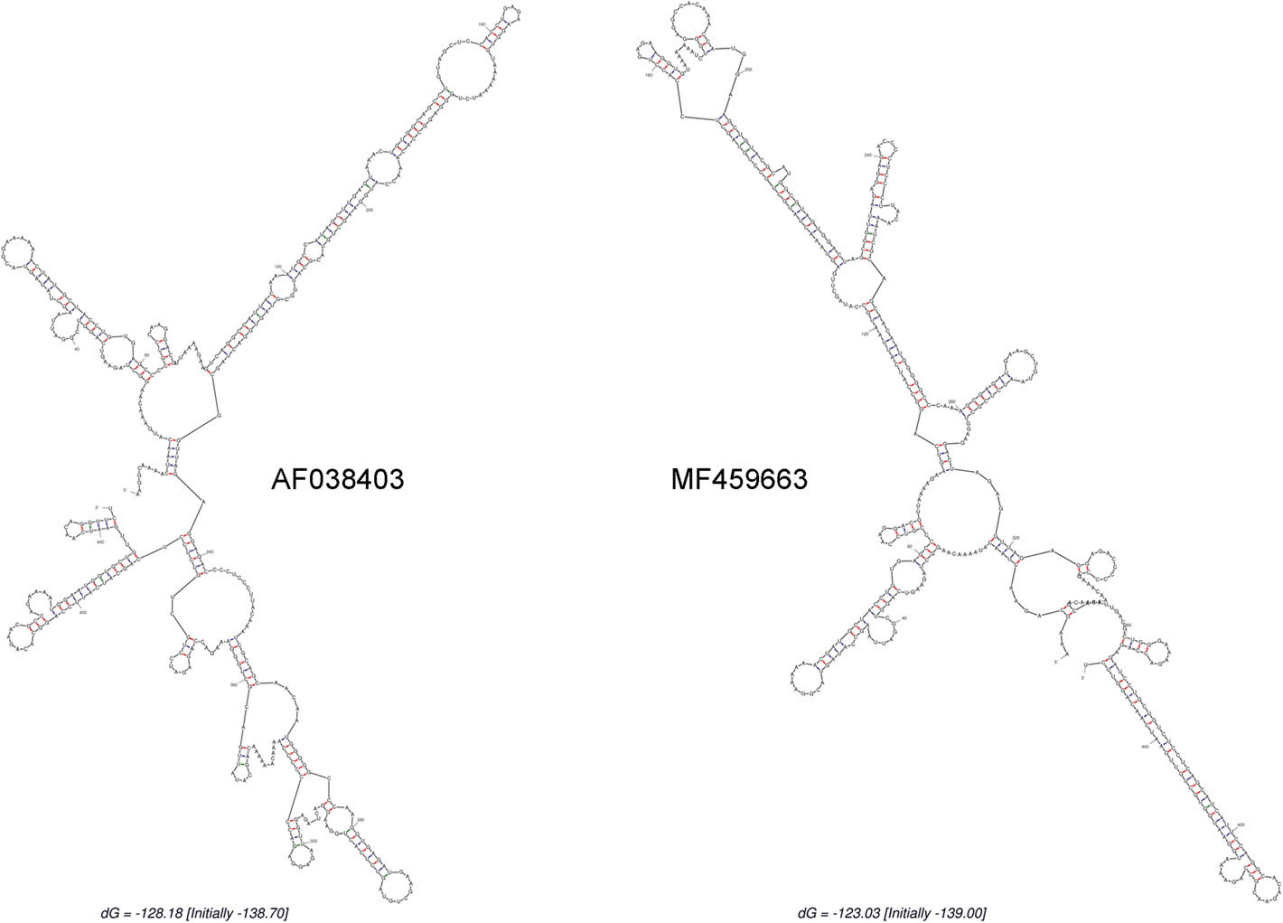
The predicted secondary structure of 3′ UTRs of the prototype and YNPE2. **Left:** the predicted secondary structure of 3′ UTR of the prototype. **Right:** the predicted secondary structure of 3′ UTR of YNPE2

The results of RNA-seq yielded 7,811,508 clean reads from 8,328,038 raw reads after removing unqualified reads. The full-length sequence of the YNPE2 strain was assembled by SPAdes software based on the RNA-seq data and the referenced genome of DENV-2 (DQ181797). The assembled full-length sequence of YNPE2 was 10,741 nucleotides (nts) with an ORF encoding a polyprotein of 3391 amino acids, flanked by 160 nts and 405 nts at the 5′ UTR and 3′ UTR, respectively. The RACE was considered a reliable method for confirming the extreme ends of RNA genome. Thus, the UTR sequences of viral genome were verified by 5′/3′ RACE and Sanger sequencing. Interestingly, the Sanger sequencing results of 5′ UTR and 3′ UTR were different from the Illumina′s next-generation sequencing (NGS). Sanger sequencing showed that the 5′ UTR and 3′ UTR were 96 nts and 452 nts, respectively (Fig. 2). Thus, based on 5′/3′ RACE and Sanger sequencing, the complete genome of YNPE2 was corrected to 10, 724 nts, flanked by 96 nts and 452 nts at the 5′ UTR and 3′ UTR, respectively. To determine the sequence identity between the YNPE2 strain and other DENV-2 strains, the complete genome sequence of YNPE2 was aligned with the prototype strain of DENV-2 (New Guinea C, AF038403) and 38 other globally representative DENV-2 strains (Additional file 1: Table S1). As shown in Table 1, the YNPE2 strain showed 89.5–99.0% sequence identity with the other strains. No insertion or deletion of nucleotides were observed in the protein-coding region when aligned with these representative strains. Moreover, the alignment results revealed that base substitutions were distributed over the whole stretch of the alignment. A single base substitution at position 54 in the 5′ UTR was observed when aligned with the prototype. As compared to the 3′ UTR of the prototype, the YNPE2 had 14 substitutions (position 10,275 [G → A], 10,279 [A → G], 10,289 [G → A], 10,321 [T → C], 10,387 [T → C], 10,389 [C → T], 10,390 [A → T], 10,413 [G → A], 10,418 [A → T], 10,553 [G → A], 10,555 [T → C], 10,570 [G → A], 10,575 [A → G] and 10,609 [A → G]), a single base (G) deletion at position 10,415 and a single nucleotide (A) insertion at position 10,455. The RNA secondary structure prediction results showed that these nucleotides substitutions in the 3′ UTR of YNPE2 led to minor change in the secondary stem loop structure as compared to the prototype (Fig. 3). The predicted RNA secondary structure of 3′ UTR of YNPE2 had fewer loops than the prototype.

**Table 1:** Nucleotide (NT) and amino acid (AA) identity of YNPE2 (MF459663) complete genome compared to representative globally diverse full-length DENV-2 sequences

Serial number	Accession number	NT identity (%)	AA identity (%)
1	EU056811	89.5	96.7
2	GQ868588	89.5	96.7
3	AY702040	89.7	96.7
4	HM582105	89.8	96.8
5	HM582108	89.8	96.9
6	HM582099	89.9	96.9
7	JN851123	91.1	97.2
8	HQ012538	92.1	97.1
9	JX470186	91.2	97.1
10	FJ898454	91.3	97.4
11	FJ850088	91.4	96.7
12	GQ252676	91.4	97.4
13	EU179858	91.5	97.0
14	EU482636	91.7	97.0
15	FJ639703	91.8	97.4
16	EU482788	91.9	97.4
17	AY702039	92.0	97.0
18	EU529695	92.2	97.1
19	GQ398271	92.1	97.1
20	GQ868540	92.2	97.1
21	FJ898450	92.3	97.3
22	GQ398264	92.3	97.3
23	DQ181801	92.4	97.4
24	AF119661	92.4	97.4
25	GQ398269	92.4	97.3
26	JF730055	94.4	98.0
27	HQ891024	94.4	97.9
28	AF204178	94.4	97.7
29	EU854293	94.6	98.1
30	AF038403	94.5	98.1
31	GQ398268	94.6	98.3
32	FJ196851	96.0	98.2
33	FJ639705	98.2	99.5
34	FJ906957	98.3	99.5
35	FJ410215	98.4	99.7
36	EU482445	98.5	99.7
37	GQ868543	98.7	99.5
38	FJ639832	99.0	99.8
39	DQ181797	99.0	99.8

### Amino acid sequence diversity

In order to characterize viral amino acid sequences, the deduced amino acid sequence of the YNPE2 strain was aligned with 39 complete genome sequences of DENV-2 representing the global diversity of DENV-2 (Additional file 1: Table S1). The sequence alignment revealed 96.7–99.8% identity among the sequences (Table 1) and amino acid substitutions throughout the complete coding region. The result indicated 66 amino acid replacements between YNPE2 and the prototype strain of DENV-2 (New Guinea C, AF038403) (Table 2). In addition, based on nucleotide and amino acid homology, two DENV-2 strains, ThD2_0078_01 strain (DQ181797) and DENV-2/TH/BID-V2157/200 strain (FJ639832), were the most closely related to YNPE2.

**Table 2:** Description of amino acid substitutions in YNPE2 compared to the prototype (New Guinea C)

Serial number	AA^a^ position (ORF)	AA position (Protein)	MF459663 (YNPE2)	AF038403 (New Guinea)
Capsid				
1	9	9	K	R
2	101	101	S	T
prM				
3	129	15	G	S
**4**	**130**	**16**	**I**	**R**
5	169	55	L	F
6	266	152	V	A
E				
7	351	71	E	D
8	363	83	K	N
***9***	***406***	***126***	***E***	***K***
10	409	129	I	V
11	421	141	V	I
12	444	164	V	I
**13**	**456**	**176**	**A**	**T**
14	506	226	K	T
15	508	228	E	G
**16**	**626**	**346**	**Y**	**H**
17	682	402	F	I
18	764	484	I	V
NS1				
19	825	50	Q	H
**20**	**855**	**80**	**A**	**S**
21	880	105	R	Q
**22**	**904**	**129**	**Y**	**H**
23	947	172	K	R
24	952	177	A	V
25	997	222	N	S
26	1022	247	L	F
**27**	**1036**	**261**	**Y**	**H**
**28**	**1039**	**264**	**I**	**T**
29	1056	281	D	E
30	1061	286	I	V
NS2A				
**31**	**1132**	**5**	**V**	**T**
32	1178	51	K	R
33	1184	57	V	M
34	**1192**	**65**	**A**	**T**
35	1247	120	V	I
36	1263	136	I	M
37	1266	139	N	K
38	**1285**	**158**	**T**	**A**
39	1308	181	L	F
40	**1316**	**189**	**T**	**A**
NS2B				
41	**1402**	**57**	**T**	**A**
42	1457	112	I	V
43	NS3			
44	1488	13	M	V
45	**1595**	**120**	**T**	**A**
46	1662	187	R	K
47	1808	333	I	M
48	1820	345	N	S
49	2024	549	K	R
50	**2081**	**606**	**A**	**T**
NS4A				
51	**2114**	**21**	**T**	**A**
**NS4B**				
52	**2262**	**19**	**A**	**T**
53	2355	112	L	F
54	2418	175	I	V
NS5				
55	**2626**	**135**	**I**	**T**
56	2792	301	R	K
57	2826	335	L	V
58	**2828**	**337**	**T**	**M**
59	2920	429	G	S
60	3122	631	N	S
61	3132	641	V	I
62	3167	676	H	S
63	3178	687	I	V
64	3189	698	K	R
65	3310	819	R	Q
66	**3356**	**865**	**A**	**T**

*Note*: The amino acid site including replacement of hydrophilic to hydrophobic or vice versa are marked in bold font. The amino acid substitution of negatively charged to positively charged amino acid is written in bold and italic font

^a^ *AA* amino acid

### Phylogenetic analysis

**Fig. 4:**
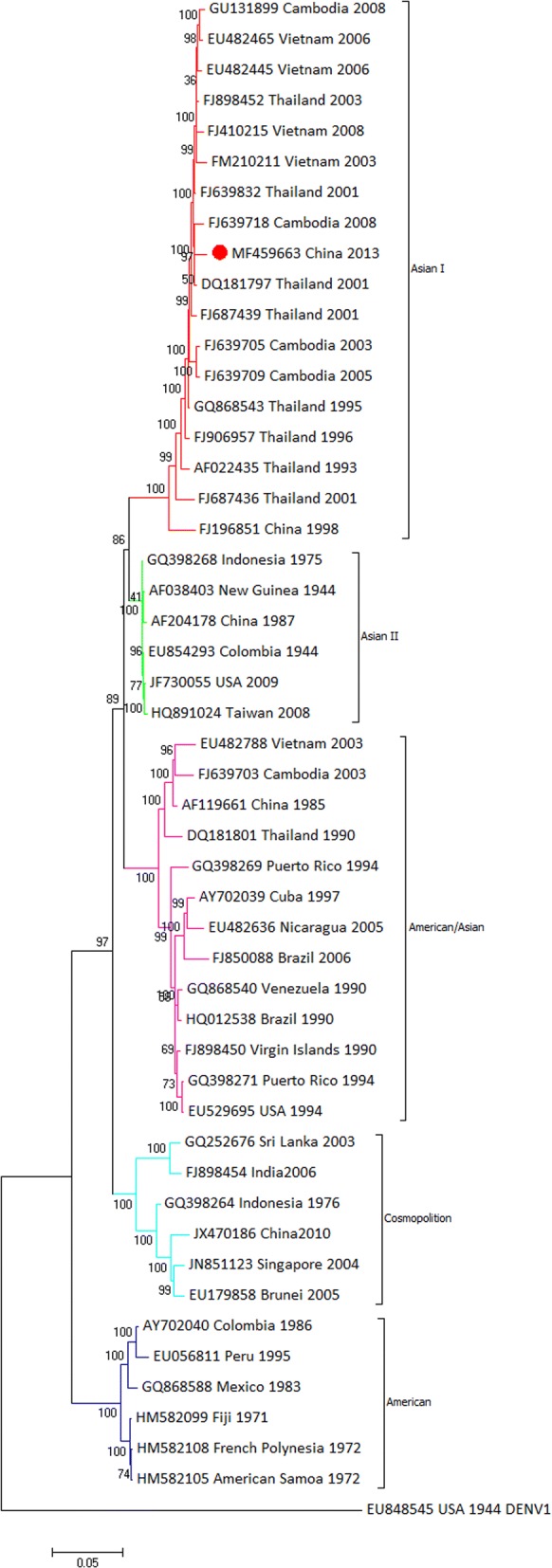
Phylogenetic tree based on the complete coding sequences of 49 DENV-2 strains. Each strain was abbreviated as the GenBank accession number followed by the country and year of isolation. The numbers at the nodes revealed bootstrap support for that node (bootstrap replications was 1000). The DENV-2 strain sequenced in this study is marked with a solid circle (●). The complete sequence of DENV-1 (US/Hawaii/1944, EU848545) was used to root the tree

**Fig. 5:**
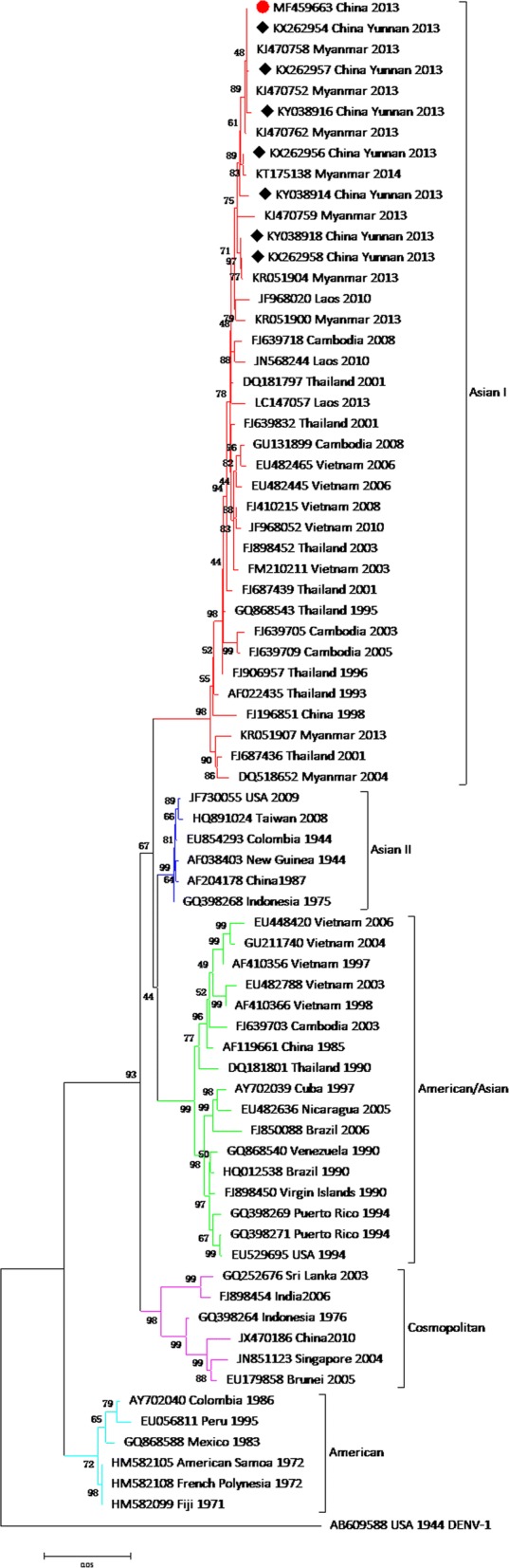
Phylogenetic tree based on 73 envelope gene sequences of DENV-2 strains. Each strain was abbreviated as the GenBank accession number followed by the country and year of isolation. The numbers at the nodes revealed bootstrap support for that node (bootstrap replications was 1000). The DENV-2 sequenced in this study is marked with a solid circle (●), and the strains isolated from Yunnan Province of China in 2013 are marked with solid diamonds (◆). The envelope gene sequence of DENV-1 (US/Hawaii/1944, AB609588) was used to root the tree

To determine the genetic relationship between YNPE2 and other DENV-2 strains isolated from geographically diverse areas, phylogenetic analysis of the whole coding sequence (Additional file 2: Table S2) and the envelope gene (Additional file 3: Table S3) were performed. The phylogenetic tree based on the complete coding sequences revealed that the DENV-2 strain belonged to the Asian I genotype along with multiple DENV-2 isolates from Vietnam, Thailand, and Cambodia. The YNPE2 strain was clustered into one group, together with Cambodia strain (FJ639718) and Thailand strain (DQ181797) (Fig. 4). The phylogenetic analysis suggested that the YNPE2 strain was likely derived from the Cambodia strain (FJ639718) or the Thailand strain (DQ181797). Moreover, the phylogenetic tree based on 73 envelope gene sequences isolated from geographically diverse areas showed that YNPE2 was clustered into a group with three Myanmar isolates (KJ470758, KJ470752 and KJ470762) and three Chinese isolates (KX262954, KX262957 and KY038916) circulating in 2013, which suggested their common origin (Fig. 5). In this study, the phylogenetic trees of the whole coding sequence and envelope gene were similar, with little diversity.

### Recombination analysis

The potential recombination events of the YNPE2 strain and other representative DENV-2 isolates (Additional file 1: Table S1) were analyzed by RDP4 software. The results of preliminary analysis revealed that four recombination events might have occurred among these DENV-2 strains (*p* < 0.01) (Additional file 4: Table S4). These recombination events seemed to be limited to some strains, accounting for 10% (4 out of 40 strains). However, no potential recombination event was detected for the YNPE2 strain within the tested isolates.

### Selection pressure analysis

To determine the influence of the selection pressure acting on complete coding region of DENV-2 isolates (Additional file 1: Table S1), an analysis based on the selection coefficient ω (dN/dS ratio) at every codon was conducted with a cutoff value (ω > 1) of positive selection. The results showed that most of the codons in DENV-2 were under negative selection. However, a total of 13 sites were verified to be under positive selection pressure through at least three out of the four approaches (Table 3). Moreover, two of these sites, positioned on NS4B and NS5 respectively, indicated important evidence of episodic positive selection through at least two different methods with a low *p*-value (*p* < 0.1). Limited evidence of positive selection was confirmed by SLAC, FEL and IFEL .

**Table 3:** Selection pressure analysis of the ORF (3391 codons) of DENV-2 using MEME, SLAC, FEL and IFEL

AA^a^ position (ORF)	MEME		SLAC		FEL		IFEL	
	ω	*p*-Value	ω	*p*-Value	ω	*p*-Value	ω	*p*-Value
prM								
248	55.584	0.062	4.778	0.445	1.525	0.277	1.123	0.278
253	> 100	0.089	2.368	0.672	0.789	0.446	< 0	1
Envelope								
408	> 100	0.093	2.309	0.712	0.824	0.406	2.171	0.333
735	> 100	0.096	1.602	0.995	0.486	9.56	< 0	1
NS1								
986	> 100	0.054	2.417	0.68	0.826	0.322	< 0	1
1010	> 100	0.017	2.431	0.664	0.713	0.416	2.098	0.305
NS2A								
1142	> 100	< 0.001	3.231	0.988	0.997	0.929	< 0	1
1195	> 100	0.093	1.685	0.946	0.576	0.819	< 0	1
NS2B								
1439	> 100	0.019	4.765	0.447	1.485	0.246	< 0	1
1459	> 100	0.014	5146	0.8	1.81	0.649	1.044	0.718
NS3								
1489	> 100	0.067	9.674	0.198	3.111	0.979	< 0	1
NS4B								
**2262**	5.444	**0.0838**	13.16	0.108	3.4	**0.088**	6.196	**0.082**
NS5								
**3310**	2.249	0.107	8.065	0.296	4.012	**0.085**	8.109	**0.058**

*Note*: Criteria to determine codons with important evidence of positive selection: *p*-value < 0.1 in MEME, SLAC, FEL and IFEL. Codons that are detected positive by at least three approaches are listed in the table. Codons with important evidence of positive selection by at least two approaches are marked in bold font

^a^*AA* amino acid

## Discussion

For human viral diseases, knowledge of the full-length genome sequence, genotype and genomic characterization of a pathogen are essential for disease surveillance, epidemiology, and vaccine development. In this study, we used complete genome sequencing and comparative sequence analysis to determine the complete genome sequence and the molecular characterization of a DENV-2 strain (YNPE2) isolated from an imported case from Myanmar. It provides critical information on the whole genome sequence and genomic characterization of DENV-2 isolates imported from Myanmar to China.

Geographically, Yunnan Province in China borders with three dengue hyper-endemic countries, including Myanmar, Laos and Vietnam. With the increase in border trade between China and Myanmar, the risk of dengue import is also increasing. In 2008, a dengue outbreak caused by imported case from Myanmar to Ruili City was monitored [21]. Forty-nine dengue patients were clinically diagnosed based on the IgM antibody and viral nucleic acid detection, of which 48 were imported from Myanmar [21]. Moreover, a recent study on dengue epidemic characterization in Yunnan also demonstrated that the imported cases from Myanmar and Laos were mainly responsible for the dengue outbreak in Yunnan [43]. Thus, the investigation of complete genome and molecular characterization of different imported DENV isolates is essential for control and prevention of DENV circulating in Yunnan. In the present study, next-generation sequencing (NGS) Illumina Hiseq 4000 was employed for whole genome sequencing of the YNPE2 strain based on its advantages of high throughput, ultra-high speed, no mark and low cost [44, 45]. The UTR sequences were confirmed by 5′/3′ RACE, which is a credible method for determining the precise UTRs of RNA genome. The 5′/3′ RACE results were inconsistent with NGS, perhaps due to some artefacts of deep sequencing or assembly protocols. Therefore, 5′/3′ RACE and Sanger sequencing are essential for determining the precise viral genome. A highly reliable full-length genome sequence of YNPE2 was obtained using NGS, 5′/3′ RACE and Sanger sequencing. The conserved UTRs at both ends of the viral genome can form stable secondary structures, which play important roles in viral translation, replication and assembly [46, 47]. Interestingly, genomic characterization analysis from this study revealed some base substitutions in the 3′ UTR. The potential biological implications of the nucleotide substitution remains unknown, but their molecular configuration was slightly changed. Further studies are needed to determine the influence of the substitution on DENV virulence and replication in host cells.

Numerous amino acid substitutions were observed in the structural and non-structural proteins of the YNPE2 strain as compared to the prototype of DENV-2 (Table 2). These amino acid substitutions may potentially influence viral fitness and virulence in host cells. Interestingly, some amino acid substitutions, such as E71D, E126K and F402I observed in the envelope protein in this study, have been confirmed in previous studies [48, 49]. These amino acid substitutions enhanced the neurovirulence of the virus. Moreover, multiple amino acid substitutions in NS1, NS2A, NS2B and NS5 proteins of YNPE2 were observed. The potential influence of these substitutions needs further investigation.

Gene recombination plays a key role in the evolution and adaptation of DENV [50]. For molecular characterization of the YNPE2 strain, we selected 39 other representative globally diverse DENV-2 complete sequences to identify potential recombination events. No potential recombination event was found in the YNPE2 strain. Perhaps, more DENV-2 complete sequences are needed to uncover any potential recombination events of YNPE2. The analysis of site-specific selection pressures for DENV-2 indicated that strong negative selection was present, which was ascribed to the evolutionary constraints of arboviruses [7]. The positive selection from host-specific immunity contributes to viral protein mutations that allow the virus to escape host immune recognition [51, 52]. In this study, two sites with important evidence of positive selection were determined. One site in NS4B was reported in an earlier study of sylvatic DENV-2 and had an inhibitory effect on the interferon signaling pathway [53, 54]. Another site in NS5 was reported for the first time in our study, and its functional importance needs further investigation.

The phylogenetic analysis, based on the complete coding sequence, showed a close relationship between the YNPE2 strain and DQ181797 (Thailand, 2001), which suggested that the YNPE2 strain very likely originated in Thailand. This conclusion was further supported by the results of the sequence and amino acid identity. A phylogenetic tree based on the envelope gene revealed different subgenotype circulating in Myanmar. Moreover, the YNPE2 strain and seven Yunnan strains are closely related to isolates from Myanmar, suggesting that the dengue outbreaks in Yunnan Province are mainly caused by imported cases from Myanmar.

## Conclusion

This study presented the first complete sequence of DENV-2 imported from Myanmar, and its molecular characterization. These results will stimulate further investigations of DENV infection, proliferation, pathogenicity, epidemiology and vaccine development.

## Additional files

Additional file 1: **Table S1.** Details of DENV-2 sequences of was used in multiple sequence alignments, recombination analysis and selection pressure analysis. (DOCX 21 kb)

Additional file 2: **Table S2.** Details of the full-length sequences of DENV-2 was used in phylogenetic analysis. (DOCX 21 kb)

Additional file 3: **Table S3.** Details of the envelope gene sequences of DENV-2 was used in phylogenetic analysis. (DOCX 23 kb)

Additional file 4: **Table S4.** DENV-2 potential recombination events obtained by RDP4 package. (DOCX 18 kb)

## Abbreviations

DENV-2: Dengue virus serotype 2
DF: Dengue fever
DHF: Dengue hemorrhagic fever
DSS: Dengue shock syndrome
FBS: Fetal bovine serum
FEL: Fixed effects likelihood
IFEL: Internal fixed effect likelihoods
MEME: Mixed effects model of evolution
ML: Maximum likelihood
NGS: Next-generation sequencing
NS: Non-structural
nts: Nucleotides
ORF: Open reading frame
PCR: Polymerase chain reaction
RDP: Recombination Detection Program
SLAC: Single-likelihood ancestor counting
UTR: Untranslated regions
