# Supplementary material for: Full-length genome and molecular characterization of dengue virus serotype 2 isolated from an imported patient from Myanmar
Source: Virol J. 2018 Aug 20;15:131. doi: 10.1186/s12985-018-1043-2 (PMC6102819; doi:10.1186/s12985-018-1043-2)
Supplement: Supplementary file 1 — Table S1. Details of DENV-2 sequences of was used in multiple sequence alignments, recombination analysis and selection pressure analysis. (DOCX 21 kb) [file 12985_2018_1043_MOESM1_ESM.docx]

**Table S1** Details of DENV-2 sequences of was used in multiple sequence alignments, recombination analysis and selection pressure analysis.

| Serial number | GenBank Accession | Country | Year | Genotype | Protein_id |
| --- | --- | --- | --- | --- | --- |
| 1 | AF038403 | New Guinea | 1944 | Asian II | AAC59275 |
| 2 | EU854293 | Colombia | 1944 | Asian II | ACH61685 |
| 3 | AF204178 | China | 1987 | Asian II | AAF18447 |
| 4 | GQ398268 | Indonesia | 1975 | Asian II | ADK37484 |
| 5 | JF730055 | USA | 2009 | Asian II | AEH59348 |
| 6 | HQ891024 | Taiwan | 2008 | Asian II | AEH59344 |
| 7 | DQ181797 | Thailand | 2001 | Asian I | ABA61176 |
| 8 | EU482445 | Vietnam | 2006 | Asian I | ACA48712 |
| 9 | FJ639705 | Cambodia | 2003 | Asian I | ACL99223 |
| 10 | FJ410215 | Vietnam | 2008 | Asian I | ACJ04257 |
| 11 | FJ639832 | Thailand | 2001 | Asian I | ACL99119 |
| 12 | FJ906957 | Thailand | 1996 | Asian I | ACQ44515 |
| 13 | GQ868543 | Thailand | 1995 | Asian I | ACW82867 |
| 14 | FJ196851 | China | 1998 | Asian I | ACN54390 |
| 15 | ***MF459663*** | ***China*** | ***2013*** | ***Asian I*** | ***ASN77915*** |
| 16 | DQ181801 | Thailand | 1990 | American/Asian | ABA61180 |
| 17 | AF119661 | China | 1985 | American/Asian | AAD18036 |
| 18 | EU482788 | Vietnam | 2003 | American/Asian | ACA49055 |
| 19 | FJ639703 | Cambodia | 2003 | American/Asian | ACL99221 |
| 20 | FJ898450 | Virgin Islands | 1990 | American/Asian | ACQ44489 |
| 21 | GQ398271 | Puerto Rico | 1994 | American/Asian | ADK37487 |
| 22 | EU529695 | USA | 1994 | American/Asian | ACA58332 |
| 23 | GQ868540 | Venezuela | 1990 | American/Asian | ACW82864 |
| 24 | HQ012538 | Brazil | 1990 | American/Asian | ADV71220 |
| 25 | GQ398269 | Puerto Rico | 1994 | American/Asian | ADK37485 |
| 26 | FJ850088 | Brazil | 2006 | American/Asian | ACO06168 |
| 27 | AY702039 | Cuba | 1997 | American/Asian | AAW31412 |
| 28 | EU482636 | Nicaragua | 2005 | American/Asian | ACA48903 |
| 29 | GQ398264 | Indonesia | 1976 | Cosmopolitan | ADK37480 |
| 30 | JN851123 | Singapore | 2004 | Cosmopolitan | AES93108 |
| 31 | EU179858 | Brunei | 2005 | Cosmopolitan | ABW06613 |
| 32 | JX470186 | China | 2010 | Cosmopolitan | AFU65934 |
| 33 | GQ252676 | Sri Lanka | 2003 | Cosmopolitan | ACS32038 |
| 34 | FJ898454 | India | 2006 | Cosmopolitan | ACQ44493 |
| 35 | AY702040 | Colombia | 1986 | American | AAW31413 |
| 36 | EU056811 | Peru | 1995 | American | ABW74620 |
| 37 | GQ868588 | Mexico | 1983 | American | ACW82878 |
| 38 | HM582108 | French Polynesia | 1972 | American | ADM26227 |
| 39 | HM582099 | Fiji | 1971 | American | ADM26218 |
| 40 | HM582105 | American Samoa | 1972 | American | ADM26224 |

*Note:* The strain sequenced in this study was marked in bold and Italic font.
