# Supplementary material for: Full-length genome and molecular characterization of dengue virus serotype 2 isolated from an imported patient from Myanmar
Source: Virol J. 2018 Aug 20;15:131. doi: 10.1186/s12985-018-1043-2 (PMC6102819; doi:10.1186/s12985-018-1043-2)
Supplement: Supplementary file 3 — Table S3. Details of the envelope gene sequences of DENV-2 was used in phylogenetic analysis. (DOCX 23 kb) [file 12985_2018_1043_MOESM3_ESM.docx]

**Table S3** Details of the envelope gene sequences of DENV-2 was used in phylogenetic analysis.

| Serial number | GenBank Accession | Country | Year | Genotype |
| --- | --- | --- | --- | --- |
| 1 | AF038403 | New Guinea | 1944 | Asian II |
| 2 | EU854293 | Colombia | 1944 | Asian II |
| 3 | AF204178 | China | 1987 | Asian II |
| 4 | GQ398268 | Indonesia | 1975 | Asian II |
| 5 | JF730055 | USA | 2009 | Asian II |
| 6 | HQ891024 | Taiwan | 2008 | Asian II |
| 7 | DQ181797 | Thailand | 2001 | Asian I |
| 8 | EU482445 | Vietnam | 2006 | Asian I |
| 9 | FJ639705 | Cambodia | 2003 | Asian I |
| 10 | FJ410215 | Vietnam | 2008 | Asian I |
| 11 | FJ639832 | Thailand | 2001 | Asian I |
| 12 | FJ906957 | Thailand | 1996 | Asian I |
| 13 | GQ868543 | Thailand | 1995 | Asian I |
| 14 | FJ196851 | China | 1998 | Asian I |
| 15 | ***MF459663*** | ***China*** | ***2013*** | ***Asian I*** |
| 16 | EU482465 | Vietnam | 2006 | Asian I |
| 17 | FJ639709 | Cambodia | 2005 | Asian I |
| 18 | FJ639718 | Cambodia | 2008 | Asian I |
| 19 | FJ898452 | Thailand | 2003 | Asian I |
| 20 | FM210211 | Vietnam | 2003 | Asian I |
| 21 | FJ687436 | Thailand | 2001 | Asian I |
| 22 | AF022435 | Thailand | 1993 | Asian I |
| 23 | FJ687439 | Thailand | 2001 | Asian I |
| 24 | GU131899 | Cambodia | 2008 | Asian I |
| 25 | DQ518652 | Myanmar | 2004 | Asian I |
| 26 | JN568244 | Laos | 2010 | Asian I |
| 27 | JF968020 | Laos | 2010 | Asian I |
| 28 | LC147056 | Laos | 2013 | Asian I |
| 29 | AF410366 | Vietnam | 1998 | Asian I |
| 30 | AF410356 | Vietnam | 1997 | Asian I |
| 31 | EU448420 | Vietnam | 2006 | Asian I |
| 32 | JF968052 | Vietnam | 2010 | Asian I |
| 33 | GU211740 | Vietnam | 2004 | Asian I |
| 34 | KT175138 | Myanmar | 2014 | Asian I |
| 35 | KJ470762 | Myanmar | 2013 | Asian I |
| 36 | KJ470758 | Myanmar | 2013 | Asian I |
| 37 | KJ470759 | Myanmar | 2013 | Asian I |
| 38 | KR051900 | Myanmar | 2013 | Asian I |
| 39 | KR051904 | Myanmar | 2013 | Asian I |
| 40 | KR051907 | Myanmar | 2013 | Asian I |
| 41 | KJ470752 | Myanmar | 2013 | Asian I |
| 42 | **KY038918** | **China Yunnan** | **2013** | **Asian I** |
| 43 | **KY038916** | **China Yunnan** | **2013** | **Asian I** |
| 44 | **KY038914** | **China Yunnan** | **2013** | **Asian I** |
| 45 | **KX262958** | **China Yunnan** | **2013** | **Asian I** |
| 46 | **KX262957** | **China Yunnan** | **2013** | **Asian I** |
| 47 | **KX262956** | **China Yunnan** | **2013** | **Asian I** |
| 48 | **KX262954** | **China Yunnan** | **2013** | **Asian I** |
| 49 | DQ181801 | Thailand | 1990 | American/Asian |
| 50 | AF119661 | China | 1985 | American/Asian |
| 51 | EU482788 | Vietnam | 2003 | American/Asian |
| 52 | FJ639703 | Cambodia | 2003 | American/Asian |
| 53 | FJ898450 | Virgin Islands | 1990 | American/Asian |
| 54 | GQ398271 | Puerto Rico | 1994 | American/Asian |
| 55 | EU529695 | USA | 1994 | American/Asian |
| 56 | GQ868540 | Venezuela | 1990 | American/Asian |
| 57 | HQ012538 | Brazil | 1990 | American/Asian |
| 58 | GQ398269 | Puerto Rico | 1994 | American/Asian |
| 59 | FJ850088 | Brazil | 2006 | American/Asian |
| 60 | AY702039 | Cuba | 1997 | American/Asian |
| 61 | EU482636 | Nicaragua | 2005 | American/Asian |
| 62 | GQ398264 | Indonesia | 1976 | Cosmopolitan |
| 63 | JN851123 | Singapore | 2004 | Cosmopolitan |
| 64 | EU179858 | Brunei | 2005 | Cosmopolitan |
| 65 | JX470186 | China | 2010 | Cosmopolitan |
| 66 | GQ252676 | Sri Lanka | 2003 | Cosmopolitan |
| 67 | FJ898454 | India | 2006 | Cosmopolitan |
| 68 | AY702040 | Colombia | 1986 | American |
| 69 | EU056811 | Peru | 1995 | American |
| 70 | GQ868588 | Mexico | 1983 | American |
| 71 | HM582108 | French Polynesia | 1972 | American |
| 72 | HM582099 | Fiji | 1971 | American |
| 73 | HM582105 | American Samoa | 1972 | American |
| 74 | AB609588 | USA | 1944 | DENV-1 |

*Note:* The strain sequenced in this study was marked in bold and Italic font. The sequences in bold referred to the sequences isolated from Yunnan Province of China.
