# Supplementary material for: Full-length genome and molecular characterization of dengue virus serotype 2 isolated from an imported patient from Myanmar
Source: Virol J. 2018 Aug 20;15:131. doi: 10.1186/s12985-018-1043-2 (PMC6102819; doi:10.1186/s12985-018-1043-2)
Supplement: Supplementary file 4 — Table S4. DENV-2 potential recombination events obtained by RDP4 package. (DOCX 18 kb) [file 12985_2018_1043_MOESM4_ESM.docx]

**Table S4** DENV-2 potential recombination events obtained by RDP4 package.

| Serial number | Recombinant DENV-2 strain [Genotype_Accession Number] | Major Parent [Genotype_Accession Number] | Minor Parent [Genotype_Accession Number] | Breakpoint start | Breakpoint end | Delection methods |
| --- | --- | --- | --- | --- | --- | --- |
| 1 | AA_GQ398269 | AA_GQ398271 | A II_AF038403 | 4529 | 6518 | RGBMCST |
| 2 | AI_FJ196851 | AI_FJ906957 | C_JN851123 | 8528 | 9614 | RGBMCST |
| 3 | AI_FJ196851 | AII_GQ398268 | C_EU179858 | 5158 | 5588 | RBT |
| 4 | AA_FJ639703 | AA_EU482788 | Unknown | 101 | 240 | GST |

*Note:* A total of 5 potential recombination events were identified by RDP4 with p-value < 0.01. The breakpoint sites were reported based on the sites in respective recombination sequence. The recombination was found by at least three of the seven detection methods named RDP, GENCONV, BootScan, MaxChi, Chimaera, SiScan and 3Seq represented as R, G, B, M, C, S and T, respectively.
